# Detection of an Extended Human Volatome with Comprehensive Two-Dimensional Gas Chromatography Time-of-Flight Mass Spectrometry

**DOI:** 10.1371/journal.pone.0075274

**Published:** 2013-09-25

**Authors:** Michael Phillips, Renee N. Cataneo, Anirudh Chaturvedi, Peter D. Kaplan, Mark Libardoni, Mayur Mundada, Urvish Patel, Xiang Zhang

**Affiliations:** 1 Breath Research Laboratory, Menssana Research Inc, Newark, New Jersey, United States of America; 2 Department of Medicine, New York Medical College, Valhalla, New York, United States of America; 3 Southwest Research Institute, San Antonio, Texas, United States of America; 4 Department of Chemistry, University of Louisville, Louisville, Kentucky, United States of America; University of Houston, United States of America

## Abstract

**Background:**

Comprehensive two-dimensional gas chromatography coupled with time-of-flight mass spectrometry (GCxGC-TOF MS) has been proposed as a powerful new tool for multidimensional analysis of complex chemical mixtures. We investigated GCxGC-TOF MS as a new method for identifying volatile organic compounds (VOCs) in normal human breath.

**Methods:**

Samples of alveolar breath VOCs and ambient room air VOC were collected with a breath collection apparatus (BCA) onto separate sorbent traps from 34 normal healthy volunteers (mean age = 40 yr, SD = 17 yr, male/female = 19/15). VOCs were separated on two serial capillary columns separated by a cryogenic modulator, and detected with TOF MS. The first and second dimension columns were non-polar and polar respectively.

**Results:**

BCA collection combined with GC×GC-TOF MS analysis identified approximately 2000 different VOCs in samples of human breath, many of which have not been previously reported. The 50 VOCs with the highest alveolar gradients (abundance in breath minus abundance in ambient room air) mostly comprised benzene derivatives, acetone, methylated derivatives of alkanes, and isoprene.

**Conclusions:**

Collection and analysis of breath VOCs with the BCA-GC×GC-TOF MS system extended the size of the detectable human volatile metabolome, the volatome, by an order of magnitude compared to previous reports employing one-dimensional GC-MS. The size of the human volatome has been under-estimated in the past due to coelution of VOCs in one-dimensional GC analytical systems.

## Introduction

In 1971, Linus Pauling reported a new technique for microanalysis of breath that revealed an unexpected finding: normal humans exhale a large number of volatile organic compounds (VOCs) in low concentrations [1]. VOC products of metabolism have subsequently been observed in animal breath [2], plants [3] and microorganisms [4], and the sum of all of the VOC products of metabolism has been termed the volatome [5]. Since Pauling’s discovery, microanalysis of human breath VOCs has led to improved understanding of physiologic processes [6] such as oxidative stress [7] and detection of new biomarkers of diseases including lung cancer [8], breast cancer [9], gastric cancer [10], uremia [11], and pulmonary tuberculosis [12].

Pauling’s human subjects exhaled through a tube chilled with dry ice in isopropyl alcohol, and the cryogenically concentrated breath VOC samples were analyzed with gas chromatography (GC). Similar techniques usually reveal around 70 different VOCs in a sample of breath [13]. However, methods for collecting, concentrating, separating and identifying breath VOCs have progressively improved during the past 40 years, and with these improvements, an increasingly complex picture of the composition of breath VOCs has emerged [6,14,15]. By the late 1990’s, one-dimensional GC and mass spectrometry (GC-MS) routinely detected approximately 200 different VOCs in a sample of concentrated human breath, mostly in nanomolar or picomolar concentrations [16]. More recently, comprehensive two-dimensional gas chromatography coupled with time-of-flight mass spectrometry (GC×GC-TOF MS) has been proposed as a powerful new tool for multidimensional analysis of complex samples with the potential to identify an even greater number of VOCs in normal human breath as well as biomarkers of diseases including asthma [17-19]. We employed GC×GC-TOF MS to explore the range of breath VOCs that could be detected in a group of healthy normal human subjects.

## Materials and Methods

### Human subjects

Breath samples were collected from a group of 34 normal healthy volunteers (mean age = 40 yr, SD = 17 yr, male/female = 19/15). All were non-smokers, and all gave written informed consent to participate in the research which was approved by the Institutional Review Board of St. Michael’s Medical Center, Newark, NJ.

### Breath VOC sample collection

The method has been described previously [20]. In summary, subjects respired normally for 2.0 min through a disposable valved mouthpiece and a bacterial filter into the breath reservoir of a portable breath collection apparatus (BCA) (Menssana Research, Inc., Fort Lee, NJ 07024). VOCs in 1.0 L alveolar breath and 1.0 L room air were captured onto separate sorbent traps containing graphitized carbon black (Supelco, Inc, Bellefonte, PA 16823).

### Sample analysis with GCxGC TOF-MS

A Unity 2 thermal desorber (Markes International Inc., DE 19807, USA) was used to purge water vapor from the sorbent traps with helium and then thermally desorb VOCs onto a cold trap for re-concentration. VOCs were desorbed from the cold trap onto the head of the primary column of a Pegasus 4D GC×GC-TOF MS system equipped with an Agilent 6890 gas chromatograph, a LECO two-stage cryogenic modulator and a secondary oven (LECO Corp, St. Joseph, MI 49085). A 30 m × 0.25 mm i.d. × 0.25 µm *d*_*f*_ Rtx-5 column (Restek Corp., Bellefonte, PA) was connected to a 2 m × 0.18 mm i.d. × 0.18 µm *d*_*f*_ Rxi-17 column (Restek Corp., Bellefonte, PA) in series and separated by the cryogenic modulator so that the non-polar primary column separated VOCs according to their boiling point and the polar secondary column in the secondary oven separated VOCs according to their polarity. VOCs eluting from the secondary column were detected with TOF MS. Helium was used as the carrier gas at 2.0 mL/min controlled via automated pressure ramp. The back inlet was set to Splitless Mode with a temperature of 200 °C for the duration of the run. We used multiple temperature ramps from 35 °C to 250 °C, during which the first and the second column have identical temperature ramp rate with the secondary oven temperature program +20 °C relative to the primary GC oven. The thermal modulator offset was +40 °C relative to the primary oven temperature. The modulation period was 5 seconds. The MS range of mass-to-charge ratio (m/z) was 35-400 and 200 mass spectra were acquired per second. The ion source chamber was held at 200 °C. The detector voltage was 1650 V with electron energy of 70 eV. The sensitivity of the system was monitored using an internal standard comprising 2 ppm of 1-bromo-4-fluoro-benzene (BFB) (Supelco, Inc, Bellefonte, PA 16823) injected on each sample tube prior to desorption.

### Determination of system repeatability

Laboratory control tubes were prepared using a mixture of acetone, anthracene, benzaldehyde, cyclohexane, limonene, toluene and n-alkanes (C5-C31) spiked onto sorbent traps. During the analysis of a large set of breath samples (not reported here), 84 of these laboratory control tubes were run, two at the beginning and two at the end of each days operation. The retention times and peak area information of each spiked-in compound are used to investigate the variations due to the analytical platform and data analysis.

### Analysis of comparison samples with 1-dimensional GC-MS

Two sequential samples were collected from the same subject and analyzed on two different instruments, GCxGC TOF-MS as described above, and 1-D GC-MS employing a previously reported method [20].

### Data analysis

LECO’s ChromaTOF software was employed to reduce the raw GC×GC-TOF MS instrument data into a list of metabolites, first by detecting peaks and then identifying VOCs by matching their mass spectral signatures to a library of mass spectra (NIST 2.0, Gaithersburg, MD 20899-1070). Peak lists obtained in different chromatograms were filtered based on retention index matching [21] and then aligned by an improved version of DISCO algorithm [22]. Low quality peaks were filtered and multiple peak entries were merged using the default values specified in DISCO. Spectral similarity was calculated using the method of Kim et al [23].

### Determination of alveolar gradients

For each breath VOC, the value of the alveolar gradient was determined as *Vb*/*I*_*b*_ -*Va*/*I*_*a*_, where *V*_*b*_= area under curve of the chromatographic peak of the VOC in breath, *I*_*b*_ = area under curve of the internal standard (i.e., BFB) peak in the same chromatogram, and *V*_*a*_ *and I*_*a*_ denote the corresponding values in the associated room air sample, respectively [20].

## Results

### Human subjects

None reported any adverse effects of donating a breath sample.

### Chromatograms

**Figure 1:**
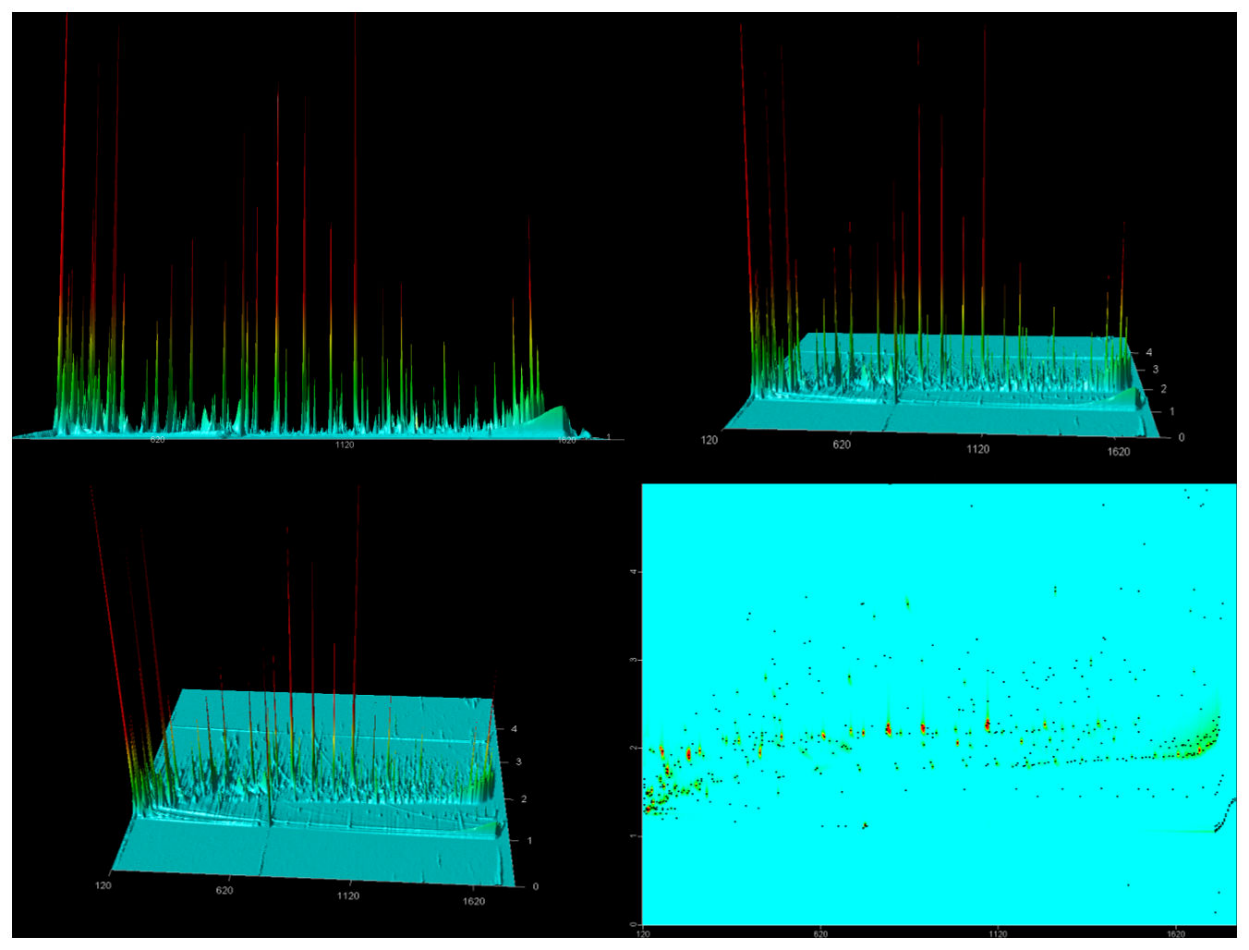
Chromatogram displaying analysis of breath VOCs in a typical normal human subject. The x-axis (horizontal) displays retention time (sec) on the non-polar primary column, and the z-axis (front to rear) displays retention time (sec) on the secondary polar column. The y-axis (vertical) represents the intensity of the peak and varies with the abundance of a VOC and the molecule specific (but not currently described) sensitivity of the method to each analyte. *Panel 1: Zero rotation about x-axis*. In this view, the z-axis is not visible, and the chromatogram resembles a conventional 1D GC MS chromatogram displaying approximately 150-200 peaks. *Panels 2 and 3: 30 and 60 degrees rotation about x-axis*. As the chromatogram rotates, peaks that appeared apparently single on the x-axis in Panel 1 are resolved into several subsidiary peaks on the z-axis. *Panel 4: 90 degrees rotation about x-axis*. Each dot represents an individual VOC in the chromatogram. TOF-MS identified approximately 2,000 different VOC peaks in this chromatogram. This provides a more sensitive depiction of the chromatographic data because it displays VOCs whose peaks are too small to be visible in the other panels. Several different categories of chemical species were observed, including terpenes, alcohols, ketones, alkanes, alkenes, esters, aldehydes, furans, benzene derivatives, and sulfides. Contour plot displays of GC×GC peaks can potentially separate breath VOCs into “chemical islands”. For example, alkanes constitute the majority of the VOCs in the oval areas outlined in the figure. Groups of similar VOCs, differing by a methyl group for example, are resolved by this technique.

Figure 1 displays a chromatogram of breath VOCs in a typical normal human subject. The x-axis (horizontal) displays retention time (sec) on the non-polar primary column, and the z-axis (front to rear) displays retention time (sec) on the secondary polar column. The y-axis (vertical) represents the intensity of the peak and varies with the abundance of a VOC and the molecule specific (but not currently described) sensitivity of the method to each analyte.

Panels 1, 2, 3 and 4 display zero, 30, 60, and 90 degrees rotation about x-axis.

**Figure 2:**
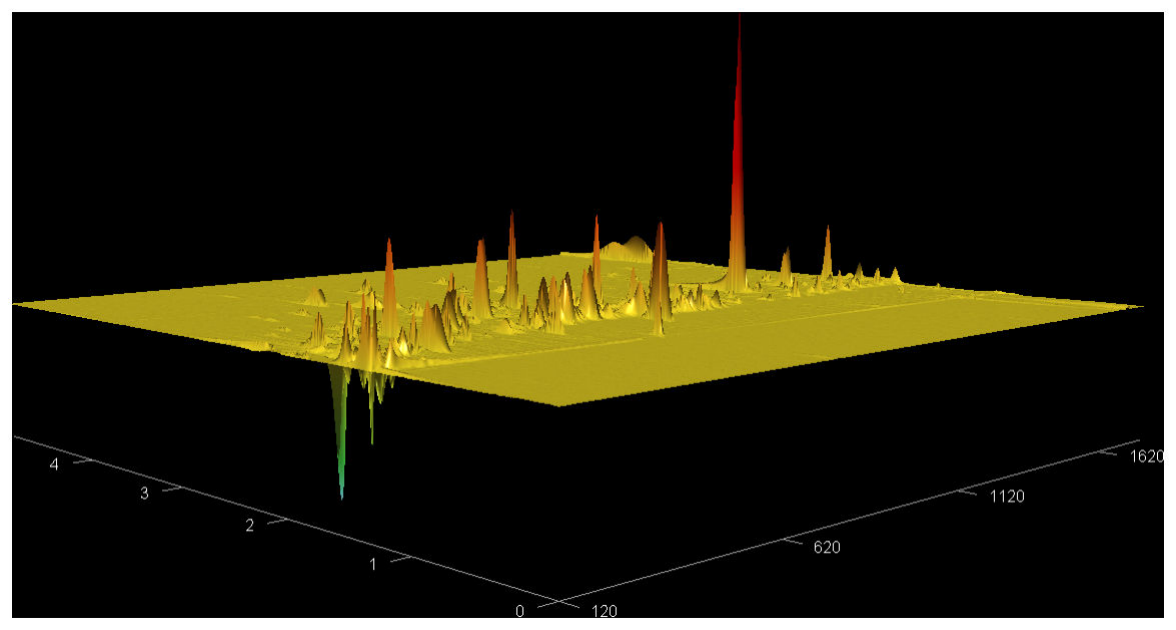
Topographical view of subtractive chromatogram displaying alveolar gradients in a typical normal human subject. Background air VOCs have been subtracted from the breath VOCs. In this view, rotated in comparison to Figure 1, the x-axis (lower right) displays retention time (sec) on the non-polar primary column, and the z-axis (lower left) displays retention time (sec) on the secondary polar column. Generally, positive peaks represent endogenous VOCs synthesized in the body and negative peaks represent ambient room air VOCs that have been cleared from the body by catabolism and/or renal excretion.

Figure 2 displays a topographical view of subtractive chromatogram of alveolar gradients in a typical normal human subject.

### Heat maps

**Figure 3:**
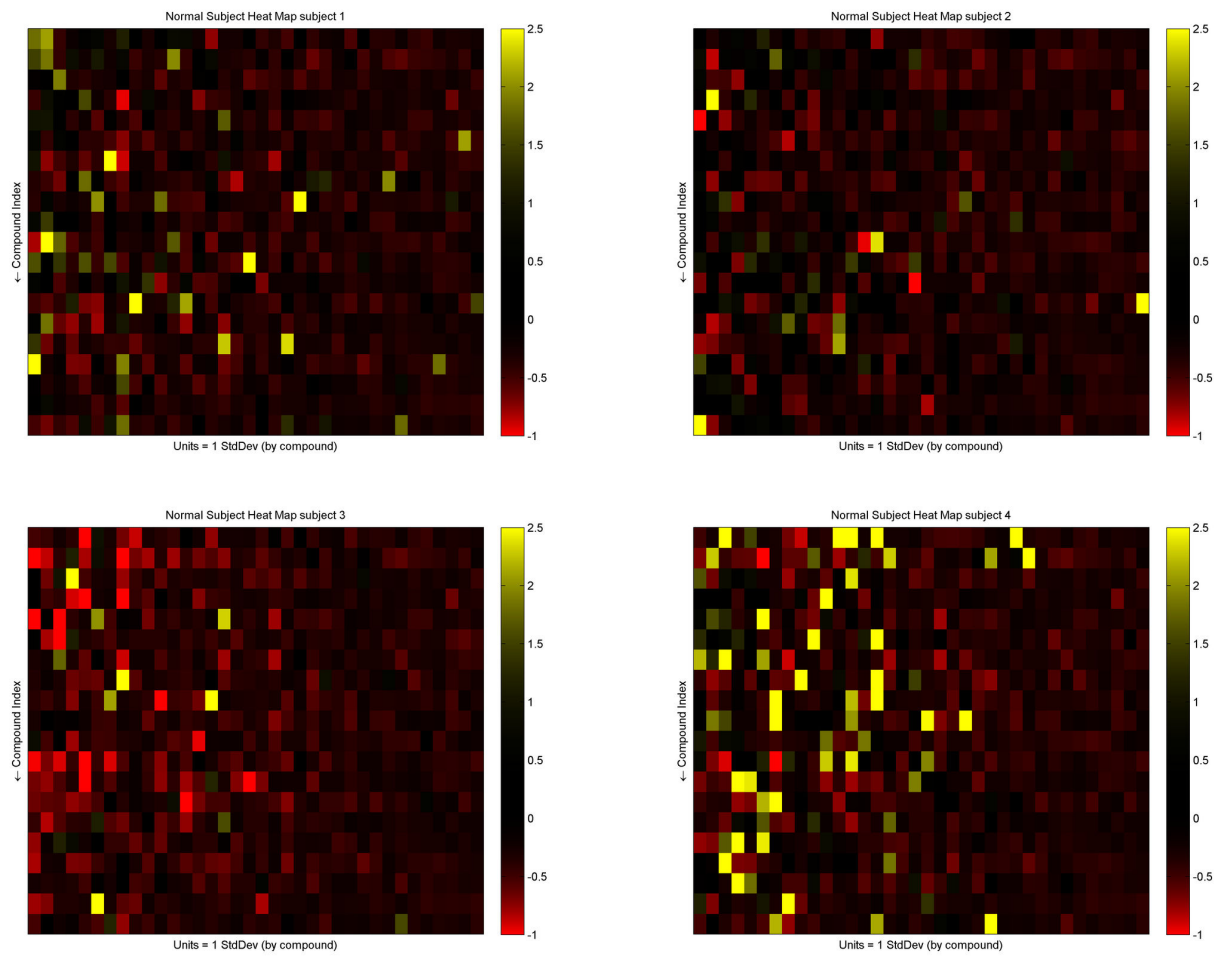
Heat maps of breath VOCs. The abundance of VOCs with an alveolar gradient greater than zero is shown in four randomly selected subjects (Figure 3). Color coding indicates the number of standard deviations by which the abundance of a VOC in an individual differs from the mean abundance in all subjects.

The mean abundance of all peaks was determined. The abundance of VOCs with an alveolar gradient greater than zero is shown in four randomly selected subjects (Figure 3).

### Analysis of comparison samples (Figure 4)

The 1-dimensional chromatogram resolved 200 peaks while the two-dimensional chromatogram (not pictured) resolved 1,016 peaks. 1-D GC-MS identified hexane at a retention time of 7.23 minutes (peak a), while 2-D GCxGC TOF-MS identified hexane at a retention time of 2.3 minutes (peak g). However, GCxGC TOF-MS identified additional peaks with the same first retention time as hexane with second retention times ranging from 1.06-1.69 seconds (peaks b-h).

**Figure 4:**
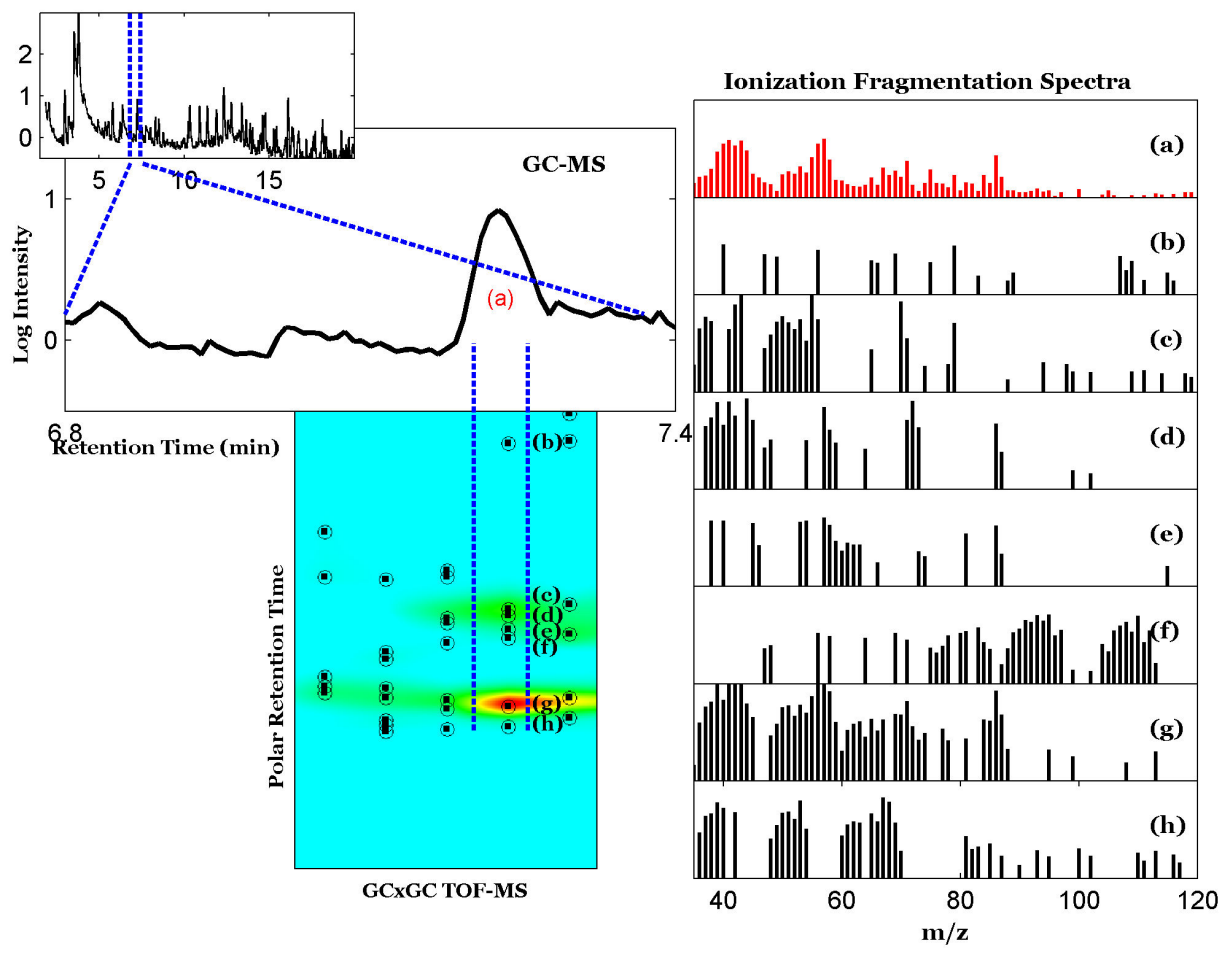
Comparison of 1D and 2D chromatograms containing hexane. The bottom panel shows the 1D chromatogram of breath VOCs in a single subject (inset) with detail around the hexane peak at 7.23 minutes (peak a). The ion fragmentation spectrum of the peak is displayed on the right in red. The top panel shows six peaks (b-h) in the 2D chromatogram that coeluted with hexane (c) on the non-polar column but with different retention times on the polar column. These peaks were identified by Chroma-TOF and the NIST library as (b) 1,3-pentadiene (c) hexane, (d) dimethyl selenide, (e) 4H-pyrazole, 3-tert-butylsulfanyl-4,4-bistrifluoromethyl- (f) butanal, (g) methyl vinyl ketone and (h) 3,5-dihydroxybenzamide. The ion fragmentation spectrum of each peak is displayed on the right. All intensities in this figure are plotted on a logarithmic scale.

### Identification of breath VOCs

Table 1 displays the most abundant VOCs observed in human breath samples ranked according to their alveolar gradient (abundance in breath minus abundance in room air) [24]. Compounds present in >90% of the normal breath samples were employed to identify the VOCs with the highest alveolar gradients.

**Table 1:** VOCs most prevalent in human breath.

**Name**	**CAS**
Toluene	108-88-3
p-Xylene	106-42-3
Benzene	71-43-2
Ethylbenzene	100-41-4
Acetone	67-64-1
Decane	124-18-5
Undecane, 2,6-dimethyl-	17301-23-4
Undecane	1120-21-4
Styrene	100-42-5
Tetradecane	629-59-4
Nonane, 2-methyl-	871-83-0
Nonane, 3-methyl-	5911-04-6
Decane, 5-methyl-	13151-35-4
Octane, 4-methyl-	2216-34-4
Cyclopropane, ethylidene-	18631-83-9
Decane, 3,7-dimethyl-	17312-54-8
Propanoic acid, anhydride	123-62-6
1-Hexanol, 2-ethyl-	104-76-7
Dodecane	112-40-3
1-Propene, 1-(methylthio)-, (E)- (isoprene)	42848-06-6
Furan, 2-methyl-	534-22-5
Cyclohexane	110-82-7
Tridecane	629-50-5
Hexane	110-54-3
Nonane, 2,6-dimethyl-	17302-28-2
2,4-Dimethyl-1-heptene	19549-87-2
Methyl vinyl ketone	78-94-4
Cyclohexene, 1-methyl-4-(1-methylethenyl)-, (S)-	5989-54-8
Heptane, 2,2,4,6,6-pentamethyl-	13475-82-6
Decane, 2,2,8-trimethyl-	62238-01-1
Hexane, 2,2-dimethyl-	590-73-8
Benzene, 1-methyl-2-(1-methylethyl)-	527-84-4
Cyclopentane, 1,2-dimethyl-, cis-	1192-18-3
Cyclooctane, 1,4-dimethyl-, cis-	13151-99-0
Decane, 4-methyl-	2847-72-5
Octane, 6-ethyl-2-methyl-	62016-19-7
1,3-Pentadiene, (Z)- (isoprene)	1574-41-0
Hexane, 3-methyl-	589-34-4
1,3,5-Trioxane	110-88-3
Benzene, 1-ethyl-3-methyl-	620-14-4
Heptane, 2-methyl-	592-27-8
Bicyclo[3.1.1]hept-2-ene, 2,6,6-trimethyl-, (ñ)-	2437-95-8
Heptane	142-82-5
2,2'-Bifuran, octahydro-	1592-33-2
Pentane, 2,3,3-trimethyl-	560-21-4
Propane, 1-(methylthio)-	3877-15-4
1-Octanol, 2-butyl-	3913-02-8
Dodecane, 2,7,10-trimethyl-	74645-98-0

VOCs in breath and air were quantified according to the ratio of their abundance to an internal standard, and ranked according to their alveolar gradient (abundance in breath minus abundance in ambient room air). Alveolar gradient varies with rate of synthesis minus rate of clearance, so that VOCs with a positive alveolar gradient generally represent products of metabolism, though they can also arise from recently ingested foodstuffs and toxins. VOCs with a negative alveolar gradient generally represent degradation of VOCs ingested from ambient room air.

### Determination of system repeatability

Variation in retention times in the two GC dimensions were small, indicating high stability of the GCxGC separation system. Specifically, 95% of all compounds had a coefficient of variation (CV) (standard deviation/mean) of less than 3% in the first dimension retention time and about 2% in the second dimension retention time. Variation in the peak area of the spiked-in compounds was larger; the range of CVs for individual components was 0.6-0.8.

## Discussion

The main new finding of this study was that GCxGC-TOF MS analysis of samples of normal human breath detected many more VOCs than have been previously reported using MS without separation [6] or one-dimensional GC MS [6,16] [25,26]. The extended size of the detectable human volatome may have resulted from improved separation of VOCs with 2-dimensional GC compared to one-dimensional GC, and the greater sensitivity of TOF-MS compared to quadrupole MS.

To illustrate the effects of coelution, we focused on the compounds that coelute with hexane (Figure 4). Where 1D GC MS identified a single peak consistent with hexane, GCxGC-TOF MS identified seven separate peaks on the second dimension, consistent with hexane and six other compounds. It is apparent that there were many ion fragments observed with 1D GC MS that did not contribute to identification of the peak as hexane. However, it would have been difficult to deconvolute these peaks into separate compounds without the aid of the polar column on the GCxGC TOF-MS. These findings support the conclusion that the size of the human volatome has been under-estimated in the past, due to coelution of VOCs in one-dimensional GC analytical systems

There are several potential applications for analysis of the human volatome with this technology, including discovery of disease biomarkers, improved detection of exposure to environmental toxins [27], and improved identification of hereditary and acquired abnormalities of metabolism. Previous studies of breath VOCs employing 1D GC MS have identified biomarkers of diseases including lung cancer [8], breast cancer [9], and pulmonary tuberculosis [12], and the application of GCxGC-TOF MS in these and other diseases offers an opportunity for improved detection of biomarkers by minimizing errors that may have been previously introduced by coelution of VOCs.

The increased size of the detectable human volatome raises a number of practical challenges for future breath research including the need for sophisticated alignment methods and for statistical tools to identify the statistically significant biomarkers of abnormal metabolism. As an example of this challenge, we note that of the 2,000 compounds identified, only 95 VOCs were detected in the breath of more than 90% of our healthy subjects and approximately 1,000 were detected in the breath of 50% or more of our subjects. These qualitative and quantitative differences between the VOCs detected in normal human samples may have resulted from inter-individual biological variation, as well as from differences in diet and in environmental exposures.

The key VOCs observed in this study were generally similar to those reported in previous studies, including acetone, isoprene and methylated derivatives of alkanes and benzene [25,26]. However, there were also notable qualitative differences that may have been due to the improved chemical resolution of the analytic tool and differences in techniques of breath VOC collection and assay which vary widely between different laboratories. Investigators are compelled to choose between several alternative options at every step in the process, including the selection of human subjects, the breath VOC collection method, the VOC trapping material, and the techniques of thermal desorption, chromatographic separation, and detection. All of these choices have the potential to bias the selection towards or away from different classes of molecules. The differences between the relative abundance of breath VOCs reported in this study and in other reports may be due to differential sensitivity, differences in ionization mechanisms amongst other causes. For example, the use of carbon black for VOC capture may have biased the sample collections in favor of relatively non-polar compounds, and the electron ionization employed in TOF MS may have yielded different patterns of fragmentation from those observed with soft ionization MS.

This highly sensitive and selective assay for breath VOCs could provide a new tool for environmental toxicology and detection of exposure to potentially hazardous volatile toxins. Also, detection of an extended human volatome may have several applications in medicine and in basic science. Previous studies of the human metabolome have focused mainly on larger molecules, and the human metabolome database with fully annotated metabolite entries had grown to more than 6800 by 2009 [28]. Detection of an extended human volatome with GC×GC-TOF MS extends the known metabolome and could provide a powerful new tool for metabolomic research and elucidation of normal and abnormal metabolic pathways. In addition, studies of the human volatome could potentially identify new volatile biomarkers of disease with greater sensitivity and selectivity, and lead to improved diagnostic technology.
